# A Therapy-Terminating Event: Programmed Death-1 Inhibitor-Induced Mucositis

**DOI:** 10.7759/cureus.29377

**Published:** 2022-09-20

**Authors:** Dawson Foster, Imad Karam, Sri Nadella, Deborah Adekunle, Margaret Meyer, Merra Rana, Joseph Sokhn

**Affiliations:** 1 Internal Medicine, St. Luke's Hospital, Chesterfield, USA; 2 Hematology and Oncology, St. Luke's Hospital, Chesterfield, USA

**Keywords:** oral cancer, mucositis, immune-related adverse effects, immuno-chemotherapy, oncology haematology, immuno-checkpoint inhibitor

## Abstract

Oral mucositis is a painful inflammatory response that can lead to infection, cachexia, and therapy termination. This immune-related adverse event (IRAE) has been well documented within the newly developing field of immunotherapy. This case series presents three patients, aged 73 to 81 years, who were undergoing treatment with programmed death-1 (PD-1) immunotherapy for cancer; each patient developed grade III mucositis, one after the fourth cycle and two after the seventh. All three patients had no prior history of oral pathology, yet each patient reported ulcerated and inflamed oral mucosa that was swollen and painful. These lesions involved various locations within the oral cavity and caused irritation to the point of dysphagia and odynophagia. Conservative treatments such as oral anesthetic and mouthwashes with antimicrobial properties had minimal effects. Each patient thereafter was started on systemic glucocorticoids in addition to the local treatments. The initiation of a systemic treatment resulted in a resolution of the oral lesions allowing each patient the option to return to their prior immunotherapy.

Mucositis is uncommon and has no standardized treatment. This case series emphasizes the debilitative result of immunotherapy-induced mucositis and illustrates the need for systemic glucocorticoids. While conservative treatments such as oral mouthwashes can be effective in treating the symptoms of mucositis, the initiation of high-dose steroids with a prolonged taper has been shown to treat the condition at its source. Early recognition of mucositis with prompt initiation of steroids has proven to be the effective mainstay treatment to relieve mucositis while limiting pauses in cancer treatment.

## Introduction

The term “chemotherapy” was first used by Paul Ehrlich in the 1930s due to his work with alkylating agents [1]. Steady gains were made in the field of oncology but with the creation of the first therapeutic monoclonal antibody, Muromonab, in 1986 the field of immunotherapy had begun [2].

Pembrolizumab is a programmed cell death-1 (PD-1) inhibitor. It was first used for melanoma and, in 2017, it was approved by the FDA for metastatic and inoperable solid tumors like non-small cell lung carcinoma, urothelial carcinoma, and many more [3]. Along with its ligands, programmed cell death ligand 1 (PD-L1), and programmed cell death ligand 2 (PD-L2), this pathway served to retain a balance between activity and quiescence as a checkpoint in DNA replication [4]. Pembrolizumab (anti-PD-1) together with nivolumab (anti-PD-1) and atezolizumab (anti-PD-L1) make up a subcategory of the checkpoint inhibitor immunotherapies [5]. Inhibition of the PD-1/PD-L1/2 pathway “uncloaks” cancer potentiating effects and induces a subsequent immune response activating T-cells, which react with proinflammatory cytokines and cytolytic molecules yielding cellular lysis and death [6]. Checkpoint inhibitors have proven to be very effective in the treatment of a wide range of neoplasms, impacting the highly mitotically active cell lines, and halting neoplastic growth. Additional cell types such as the gastrointestinal mucosa, and more specifically the oral mucosa, are highly susceptible to checkpoint inhibitors [7]. This mechanism leads to adverse outcomes.

While uncommon, side effects such as colitis, interstitial pneumonitis, rash (and other dermatological reactions), transaminitis, endocrinopathies, and mucositis have been reported [8-16]. While any portion of the gastrointestinal tract may be affected, the involvement of the oral cavity has garnered specific attention due to the severity of pain, its ability to hinder oral intake, and the increase in the likelihood of infection in an already at-risk population [17]. The treatment of mucositis is variable and unrefined. Multiple treatment approaches have been used with many supporting therapies. While secondary causes are routinely examined, the mainstay treatment of mucositis should be early initiation of systemic corticosteroids as well as a cessation of the offending agent [18]. Mucositis is becoming more common without a standardized treatment. In this report, we present three cases of oral mucositis that developed after treatment with immunotherapy and discuss how these adverse events were successfully managed.

## Case presentation

Case 1

**Figure 1:**
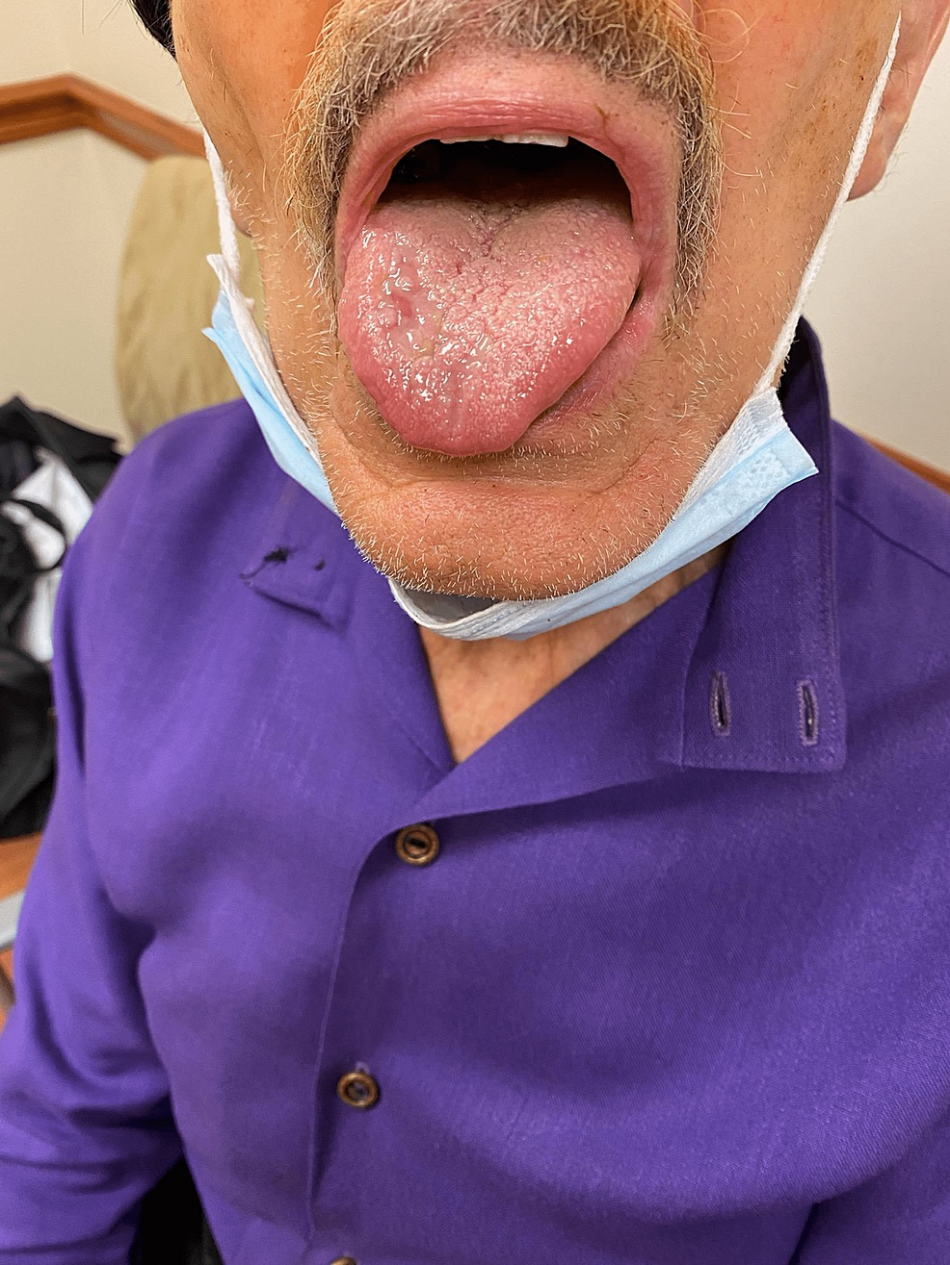
Mucositis initial presentation

**Figure 2:**
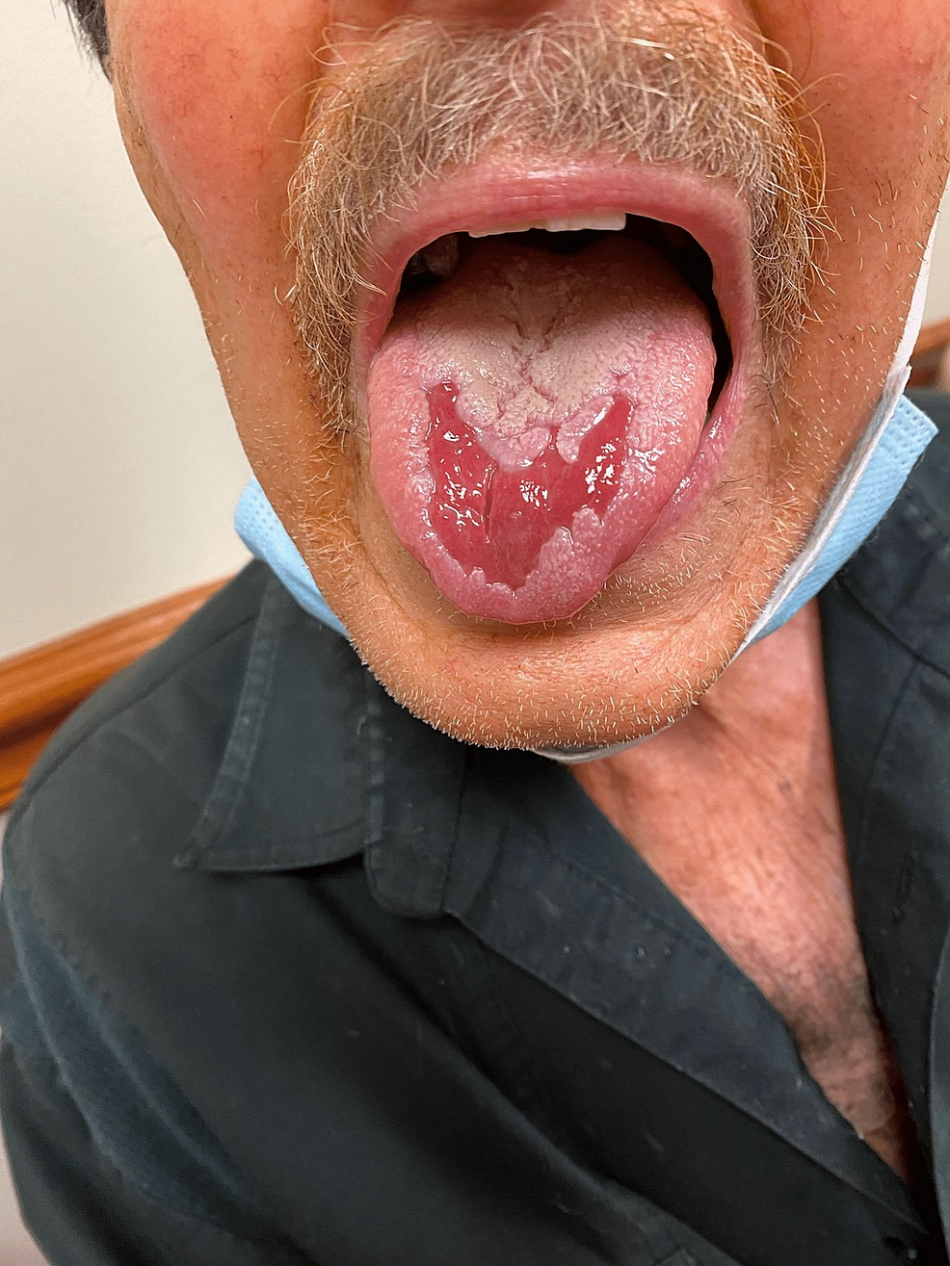
Mucositis prior to initiating conservative treatment

We present an 82-year-old male with a history of recurrent high-grade invasive papillary urothelial carcinoma with an inverted growth pattern. The carcinoma extended through the lamina propria and into the subepithelial connective tissue and was initially treated with two resections of the bladder. The patient completed 20 cycles of radiation, after which he was started on fluorouracil (5-FU) and mitomycin-C. The specimen was positive for tumor protein 63 (p63) but negative for synaptophysin and NKX3.1. The patient was started on pembrolizumab 200 mg IV every three weeks. He managed the first seven cycles well. However, after the seventh cycle, the patient developed dysphagia, loss of appetite, and painful oral lesions that were thought to be oral thrush and was treated with benzocaine lozenges and fluconazole with little to no effect (Figure 1). Right before the patient's eighth treatment, he developed worsening oral ulceration, dry mouth, and severe pain (Figure 2).

**Figure 3:**
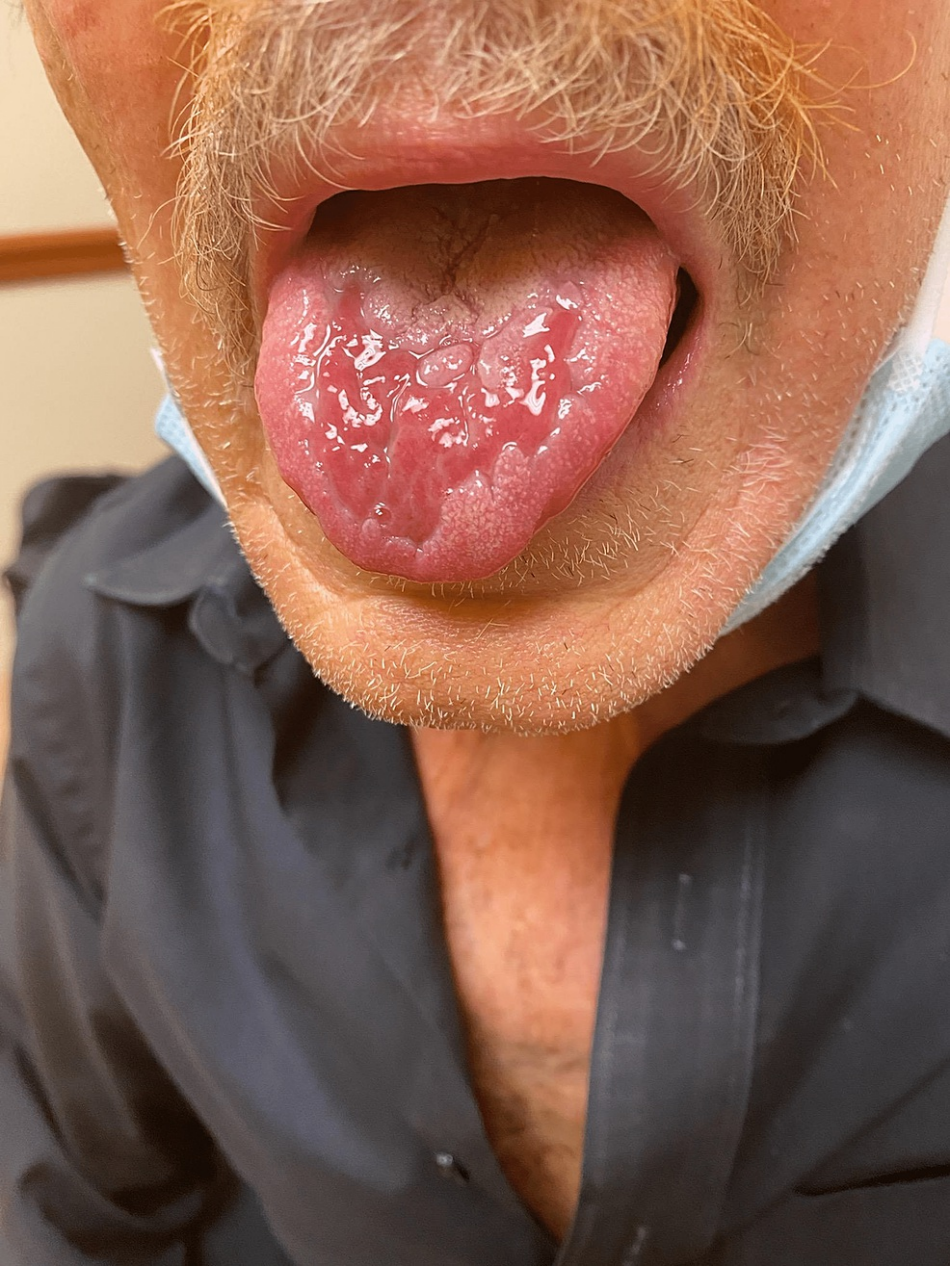
Mucositis after three weeks of conservative treatment

The patient was started on Mary’s magic mouthwash and baking soda rinses. After three weeks of worsening mucositis, the patient was hospitalized and started on IV methylprednisolone 80 mg every 24 hours, nystatin swish and swallow, fluconazole IV 200 mg every 24 hours, and Biotene (Figure 3). He was transitioned to 60 mg of oral prednisone and tapered down by 10 mg every seven days. The patient finished the given taper but required additional prednisone due to lingering ulcerations. He initially started at 40 mg of prednisone but was quickly escalated to 60 mg. After a month, the patient was slowly tapered down by 10 mg weekly without a recurrence requiring a total of nine weeks of systemic steroids. The patient was advised to continue swish and spit medications along with doxepin for prophylaxis. The patient continues to be mucositis free.

Case 2

We present a 73-year-old female with severe pain. The patient was positive for a 10-pound weight loss, diffuse tenderness in the lumbar region, paraspinal muscle tenderness, and a prior diagnosis of large cell neuroendocrine carcinoma. Palpation of the thoracic spine revealed moderate tenderness and she was unable to sit upright during the examination due to significant pain.

Laboratory studies were significant for normocytic anemia, slight leukocytosis, hyponatremia, hypochloremia, and a decrease in total protein. The abdominal computed tomography (CT) scan was significant for age-indeterminate compression deformities of lumbar 1, lumbar 3, and lumbar 4 vertebral bodies. Magnetic resonance imaging (MRI) of the lumbar spine further classified these deformities as compression fractures. Newly diagnosed lumbar compression fractures were not noted on the most recent imaging from 2019. A marrow displacing lesion was suspected. On further workup, a chest CT was performed, and a left upper lobe pulmonary nodule was identified as well as mediastinal and left hilar adenopathy. Full body positron emission tomography-computed tomography (PET-CT) showed an increase in uptake in the 12-millimeter nodule, intense activity, and enlargement of the left suprahilar, mediastinal lymph nodes, and multiple hepatic and skeletal foci were noted.

The patient was scheduled for bronchoscopy to further evaluate the pulmonary nodule. Despite the prior diagnosis of large cell neuroendocrine carcinoma that had diffuse chromogranin expression, this specimen had only weak partial expression of synaptophysin and a negative CD45, which led to a diagnosis of neuroendocrine carcinoma favoring small cell carcinoma. Carboplatin AUC5 plus etoposide 100 mg was started with atezolizumab 1200 mg every 21 days. After the patient's fourth cycle of immunotherapy, the patient developed what appeared to be oral thrush along with painful oral sores. This severe pain inhibited the patient’s oral intake. The patient was initially started on Mary’s magic mouthwash, Biotene mouthwash, and baking soda rinses.

Despite two weeks of local therapy, the worsening mucositis symptoms led to his fifth cycle of chemotherapy and immunotherapy being held. The patient presented to the emergency room and was started on a 4 mg regimen of intravenous dexamethasone to be completed over a five-day period. Symptoms improved and she was transitioned to 60 mg of oral prednisone to be tapered down by 10 mg every seven days. Once completed, her mucositis had resolved. However, with the immunotherapy on hold, a follow-up CT scan showed near complete atelectasis of the left upper lobe, an irregular narrowing of the left upper lobe bronchus, bilateral pulmonary infiltrates, and multiple hepatic metastases were identified. The patient’s cancer had worsened, and she was no longer able to undergo systemic chemotherapy. While immunotherapy was plausible, the patient and family transitioned to hospice care.

Case 3

A 73-year-old female with a history of non-small cell lung cancer and ongoing tobacco use underwent surveillance CT. The scan was remarkable for a subcarinal mass that measured approximately 4.0 x 2.7 cm in size, mediastinal lymphadenopathy, and a 2.3 x 1.5 cm precranial lymph node, which was not present in past lung screenings. The patient’s tissue biopsy was poorly differentiated pulmonary adenocarcinoma and with further workup of the tumor, genetics revealed high expression for PDL-1 (98%, 3 plus intensity). The patient was diagnosed with stage four non-small cell lung carcinoma favoring adenocarcinoma. 

Given the patient’s poor performance status and comorbidities, chemotherapy was not recommended; however, with her high PDL-1 positivity, the patient was recommended to start pembrolizumab as a monotherapy. The patient was started on pembrolizumab every 21 days at a dose of 200 mg. With continued treatment of pembrolizumab, a repeat CT showed a good response within the subcarinal mass and lymph nodes while mediastinal lymph nodes and hepatic metastasis were noted to be unchanged. After the sixth cycle of pembrolizumab, she started to develop significant blistering within the oral cavity along with multiple caries and a broken tooth. The patient was started on Mary’s magic mouthwash, baking soda water rinses, and acyclovir with no effect. The mucositis did not improve after two weeks of local treatment. She was subsequently started on oral prednisone 60 mg every 24 hours. The patient received her seventh cycle of pembrolizumab once the mucositis began to resolve. She was transitioned to oral prednisone swish and spit solution. However, once transitioned to the oral solution the patient’s mucositis reemerged. The eighth cycle of pembrolizumab was held, and the patient was started on prednisone 60 mg every 24 hours, tapering down by 10 mg every week. Once completed, the patient continued her immunotherapy without any return of mucositis.

## Discussion

Pembrolizumab has greatly changed the treatment of multiple forms of cancer are treated and has a well-accepted safety profile [19]. While impressive, the treatment is not free of adverse events. Mucositis is an uncommon occurrence but its manifestation increases the risk of treatment termination, which only becomes more likely with dysphagia and inflammation affecting oral intake. The World Health Organization (WHO) has since classified mucositis according to the degree of mucositis and severity of symptoms (Table 1) [20]. Localized therapies offer symptomatic relief, but the initiation of steroids provides definitive resolution.

**Table 1:** WHO grading scale Source: WHO Handbook for Reporting Results of Cancer Treatment [20]; with copyright permission from the World Health Organization (WHO)

Grade	Description
0 (None)	None
1 (Mild)	Oral Soreness, Erythema
2 (Moderate)	Ulcers, Erythema; Solid Diet Manageable
3 (Severe)	Ulcers; Liquid Diet Manageable
4 (Life-Threatening)	Ulcers; Oral Intake Not Possible

As it is likely that the checkpoint inhibitor immunotherapy has been held due to this IRAE, it is even more important to treat the problem effectively in hopes of resuming treatment. As with our cases, initiation of oral prednisone at 60 mg per day and tapering down by 10 mg per week is an effective treatment that provides adequate time for most of the symptoms to resolve. It was only after the initiation of steroids that significant improvement was noted. While rinses and pain relief were important, the mainstay treatment was systemic steroids.

As seen in one of the cases, recurrence can be a problem. In the case of recurrences, the priority shifted from prednisone 60 mg with a weekly tapering to a sustained four weeks of 60 mg followed by a weekly 10 mg reduction until a 10 mg dose was achieved; at that time 5 mg was given for an additional week prior to the prednisone discontinuation. Tapering should be individualized to the patient’s symptomatic improvement with hopes of not drawing out the treatment pause.

Local corticosteroid therapy has been demonstrated as beneficial within the normal population and in patients receiving immunosuppressive therapy [21,22]. Yet, if the local treatment does not induce a therapeutic response the addition of a systemic therapy starting at 60 mg and tapering by 10 mg weekly should be initiated.

Supplementary therapy such as Mary’s magic mouthwash, baking soda rinses, and additional pain management therapies should be used to support the mainstay treatment [23,24]. While it is unclear as to whether they aid in the overall treatment, these therapies provide much-needed symptomatic relief. Mary’s magic mouthwash usually contains an antihistamine, an antacid, an antifungal, and a local anesthetic to provide pain relief, along with anti-microbial, and anti-inflammatory to the applied mucosa. The baking soda and Mary’s magic mouthwash also provide a rinsing effect that helps remove food and cellular debris. However, due to most facilities having varying formulations, any attempt to standardize Mary’s magic mouthwash continues to be a difficult feat [25]. Additional pain-relieving therapies such as topical oral gel or systemic medication can and should be added for breakthrough pain (Table 2).

**Table 2:** Treatment recommendation

Systemic Treatment:	Local Treatment:	Symptomatic Management:	Other Prophylaxis:
Oral prednisone or IV dexamethasone transitioned to an oral prednisone tapper if severe	Cortical steroid washes	Mary’s magic mouthwash, baking soda rinse, and oral gel opioids	Antibiotics, antifungals, and antivirals

Focus should also be placed on gaining adequate nutritional supplementation. Cancer patients not only require additional calories for their higher-than-normal metabolic demand and high cell turnover, but they also need additional substrates for wound repair. Identifying patients at risk for nutritional deficiencies and providing a framework to combat this should not be ignored. 

While associated with immunotherapy, mucositis is not the only diagnosis that appears as a painful oral lesion. Steps should be taken to rule out other causes such as herpes simplex virus (HSV), human immunodeficiency virus (HIV), autoimmune conditions (e.g. systemic lupus erythematosus, Sjogren's syndrome), and local trauma. These conditions should be included in the initial differential diagnosis. Workup such as fourth-generation tests, HSV serology/culture, antiphospholipid antibodies, anti-dsDNA, anti-Smith, anti-Sjogren's syndrome-related protein type A (Anti-SS-A), anti-Sjogren’s syndrome-related protein type B (Anti-SS-B), and antinuclear antibodies (ANA) should be performed if consistent with a patient's family and personal history.

While the initiation of systemic steroids is beneficial in the treatment of SLE and Sjogren’s syndrome, its use in HSV and HIV provides little benefit and possibly worsen the severity. Anti-viral medication such as valacyclovir or acyclovir could be started initially to cover a wide range of conditions. Treatments for different conditions associated with mucositis are listed in Table 3.

**Table 3:** Differential diagnosis PCR: polymerase chain reaction; ANA: antinuclear antibody; RA: rheumatoid arthritis

Differential Diagnosis	Workup	Treatment
Mucositis	History taking and diagnosis of exclusion	Systemic steroids with supplementary care
Herpes simplex	PCR and/or viral culture	Acyclovir
Human immunodeficiency virus	4th generation	Retroviral
Autoimmune	ANA, RA, Jo 1, dsDNA, Smith	Systemic steroids and Immunosuppressive medication
Microbial Infection	Sputum culture, gram stain, blood cultures, fungal cultures	Appropriate broad-spectrum antimicrobials for bacteria and fungi if suspected

## Conclusions

While rare, mucositis can halt the life-saving PD-1/PDL-1 therapy providing the cancer time to further grow and divide. Local therapy can be initiated but its effects appear to alleviate the symptoms and not the cause. Quick initiation of systemic steroids with a modest taper is shown to be effective while avoiding wasted time with conservative rinses. Rinses and additional therapies can and should be initiated if medically indicated but quick initiation of systemic steroids appears to be the most effective treatment modality for mucositis. The quicker the systemic therapy can resolve the IRAE, the sooner the patient can restart their life-saving immunotherapy.
